# Association between perceived stress and history of intimate partner violence throughout life and during the COVID-19 pandemic

**DOI:** 10.3389/fpubh.2023.1330451

**Published:** 2023-12-18

**Authors:** Loys Lene da Costa Siqueira, Karla de Melo Batista, Franciéle Marabotti Costa Leite

**Affiliations:** ^1^Graduate Program in Collective Health, Department of Nursing, Health Sciences Center, Federal University of Espírito Santo, Vitória, Espírito Santo, Brazil; ^2^Department of Nursing, Health Sciences Center, Federal University of Espírito Santo, Vitória, Espírito Santo, Brazil

**Keywords:** violence against women, domestic violence, intimate partner violence, COVID-19, stress psychological, perceived stress

## Abstract

**Introduction:**

Intimate Partner Violence (IPV) is a significant public health issue, characterized by being a highly stressful experience for its victims. The relationship between IPV and stress creates a harmful cycle with broad health implications, affecting individuals and society at large. Despite its significance, there's a noticeable lack of research on this topic, especially regarding IPV throughout one's life and during the pandemic.

**Objective:**

To verify the association between perceived stress and the history of intimate partner violence throughout life and during the COVID-19 pandemic.

**Materials and methods:**

Analytical cross-sectional epidemiological study with a sample of 1,086 women. Sociodemographic information and violence history, assessed using the World Health Organization Violence Against Women (WHO VAW STUDY), along with perceived stress measured by the short version of the Perceived Stress Scale (PSS), were collected. The sampling process unfolded through multiple stages. For bivariate analyses, the *t*-test and ANOVA were performed, whereas for multivariate analyses simple and multiple linear regression were performed. The software Stata^®^ version 15.1 and R^®^ were used.

**Results:**

Women who reported having suffered intimate partner violence throughout their lives had higher means of stress (18.49), with an average increase of 4 points without adjustments and 3.5 points after adjustments for sociodemographic variables. Similarly, during the COVID-19 pandemic (19.01), stress increased by an average of 3.3 points, which was reduced to 2.8 points after adjustments.

**Conclusion:**

The results indicate an association between intimate partner violence and an increase in women's perceived stress, both throughout life and during the pandemic. The importance of preventive approaches, promoting gender equality and preventing IPV from the early stages of life is highlighted. In addition, they underscore the urgency of evidence-based interventions of a comprehensive nature to deal with this complex issue in a careful and effective manner. The cross-sectional nature of this study limits the inference of causality, and an additional limitation is acknowledged concerning information bias. This bias relates to the multifaceted issues surrounding the concept of violence, potentially influencing the accuracy of participants' information and complicating the measurement of violence.

## 1 Introduction

Intimate Partner Violence (IPV) is one of the most frequent forms of abuse committed against women. It is also identified as an important global public health concern. This form of violence is characterized by harmful physical, sexual, and/or psychological behaviors, and may even involve economic abuse and control over the victim (1). The World Health Organization (WHO) findings indicate that around 1 in 3 (30%) of women worldwide have faced intimate partner violence during their lifetime. Furthermore, globally, intimate partners account for up to 38% of all homicides targeting women (2). Similarly, in Brazil, IPV is also a concern, with 23% of women being subjected to physical and/or sexual violence by an intimate partner throughout life (3). Additionally, a study conducted in Vitória, Espírito Santo (ES), on IPV throughout life reported that 57.6% of women were victims of psychological violence, 39.3% of physical violence, and 18% of sexual violence (4).

We should also consider the scenario of the Corona Virus Disease 2019 (COVID-19) pandemic, which began in 2020. As pointed out in the literature, during this period, violence against women increased, largely due to the necessary control measures, such as social isolation, which significantly impacted women (5). Thus, violence against women remains a latent threat to both public health and women's health and wellbeing during times of crisis. The threat of violence faced by women and their children in emergency situations, such as the COVID-19 pandemic, cannot in any way be underestimated (6).

Considering this, note that IPV has wide-ranging negative consequences affecting physical, sexual/reproductive, mental, and behavioral health (1), and representing a highly stressful experience for its victims (7). Stress is an inevitable consequence of this experience for women (8), and it can persist or worsen even after the relationship ends (9). Stress refers to the adaptive biological and psychological changes that occur in response to external demands (10). Although the literature highlights short-term stress as positive and adaptive, increasing performance and improving immune function (11), in contrast, the long-term stress response is related to dysregulation of the immune system (12).

Chronic and unstoppable stress can result in damage to physical and mental health, such as anxiety disorders, depression, cardiovascular diseases, obesity, metabolic syndrome, type 2 diabetes mellitus, atherosclerosis, neurovascular degenerative disease, osteopenia, osteoporosis, and insomnia (13). Note that the theory of stress, coping, and adaptation of Lazarus et al. (14), presents stress as the consequence of perception of individuals that the demands are superior to their ability to manage them. The stress related to IPV is unique, especially due to the so-called cycle of violence that occurs in these experiences (7). Stress perception is a widely used measure to interpret or evaluate the psychological component in the response to stressors (15), including IPV (7).

Understanding stress in the most diverse sociodemographic, cultural, and social groups contributes to preventing adversities directly related to it and to other broad health problems in the world. Thus, investigating the way people perceive stressful situations in their lives is essential for computing psychological stress in health and disease around the world (16). Also, few studies, especially in Brazil, address the relationship between stress and IPV. Therefore, this cross-sectional epidemiological study, considering sociodemographic information, aimed to verify the association between perceived stress and the history of intimate partner violence throughout life and during the COVID-19 pandemic. We screened for a history of violence using the World Health Organization Violence Against Women (WHO VAW STUDY), and the short version of the Perceived Stress Scale (PSS) was employed to assess perceived stress.

## 2 Literature review

We recognize that the biology of stress is not merely an “emergency system” but rather a continuous system in which the body and the brain adjust to our routine experiences, regardless of whether we perceive them as stressful or not. These experiences encompass our adherence/lack of adherence to our circadian cycle, whether we are introverted or extroverted, and if we live in a noisy and insecure environment or have access to nature and sources of peace and tranquility (17, 18).

In common terms, “good stress” refers to short-duration experiences controlled by the individual, generating positive feelings and a sense of accomplishment. On the other hand, “bad stress” or simply “being stressed” is the opposite, associated with experiences beyond the individual's control, often prolonged, recurring, emotionally draining, and physically exhausting or dangerous (19). This type of chronic and uncontrollable stress is not only negative but can become toxic, resulting in prolonged physical and mental health damage (17, 18). It is observed, therefore, that stress arises from the interaction of the individual with the environment, representing a globally significant health issue (20).

The health consequences of exposure to IPV are vast. The hypothetical pathways through which intimate partner violence leads to distinct forms of morbidity and mortality include the direct route of violence resulting in injuries and death (21). Acute or immediate physical injuries, such as bruises, abrasions, lacerations, punctures, burns, and bites, as well as fractures and broken bones or teeth, are highlighted. Furthermore, there are more severe injuries with the potential to lead to disabilities, involving body parts such as the head, eyes, ears, chest, and abdomen, and long-term health problems, frail health status, and death, as related to Acquired Immunodeficiency Syndrome (AIDS) or feminicide (22).

Beyond the direct pathway, there is a more indirect route mediated by stress responses. Complex biological mechanisms connect exposure to violence to different adverse health outcomes through neural, neuroendocrine, and immune responses. Brain regions such as the hippocampus, amygdala, and prefrontal cortex undergo structural changes that have implications for mental health and cognitive performance. These alterations can result in mental disorders, somatoform disorders, or chronic pathologies, as well as other physical conditions (21). As mentioned earlier, stress is an inevitable consequence of IPV in women (8).

However, there is a shortage of studies on the relationship between the perception of stress and exposure to IPV, especially in the Brazilian context.

According to Lazarus et al.'s (14) theory of stress, coping, and adaptation, stress is a consequence of individual perception, considering subjectivity. This cognitive theory is grounded in evaluation, where the personal meaning (primary appraisal) of an event (stress) for the individual is assessed. Coping options are then considered, including the use of social support. Based on these assessments, the event is categorized as harm, threat, or challenge. Coping can be problem-focused or emotion-focused, and the outcome of this event can be favorable or unfavorable, resulting in positive emotion or distress, respectively (14, 23, 24).

It is inevitable to consider that there are various human experiences that can lead to stress, encompassing a wide variety of aspects and situations (25). However, stress related to IPV is unique, especially due to the cyclical nature of violence, characterized by a period of progressive tension followed by an act of violence, then a relatively calm phase, and again followed by progressive tension (7, 26). Other elements of the IPV experience, such as intensity, duration, the victim's perception of their situation, coexistence with the aggressor, the presence of other concurrent stressors, and post-traumatic symptoms, should be considered (7).

Survivors of IPV describe it as a chronic, overwhelming, emotional, and personal experience, standing out as a unique stressor in their lives. They face various challenges and obstacles in dealing with violence and associated stress, including difficulties related to the partner, lack of resources and limited support, a history of abusive relationships and other experiences of abuse, reluctance to label IPV as abuse, barriers in disclosing the incident, influence of personal and religious beliefs, and the presence of children (27).

Longitudinal data collected from a sample of 815 married women in rural Pakistan, 12 months postpartum, revealed that 8.5% of women reported experiencing physical IPV, 25.7% psychological IPV, and 25.1% sexual IPV in the last year, with 42.6% experiencing some form of IPV. Exposure to any form of IPV in the previous year (vs. none) and greater severity of IPV were associated with 3.43 and 2.57 points, respectively, of an increase in perceived stress. Physical, psychological, and sexual IPV, along with their relative severities, were independently associated with increased perceived stress (28).

Another study conducted with young adults in South Africa, investigating the association between IPV, psychosocial, sexual health, and gender-specific perceived stress, revealed that 60% of the participants were women. Higher perceived stress was observed among young women compared to young men. Additionally, young women who were victims of IPV exhibited higher levels of perceived stress (29).

A multiple mediation analysis involving 7,392 women, examining the impact of IPV, depressive symptoms, alcohol dependence, and perceived stress on the risk of cardiovascular disease over 30 years among young adult women (aged 24 to 32 years), revealed that 15% had experienced some form of IPV in the last year. Participants had, on average, moderate levels of perceived stress, few depressive symptoms, and little or no alcohol dependence. However, those exposed to IPV in the last year had statistically/significantly higher levels of perceived stress, an increase in depressive symptoms, and a higher risk of cardiovascular disease over 30 years (30).

The exposure to acute and chronic psychological stress has been associated with pathological conditions for both men and women; however, women are more vulnerable to the detrimental effects of stress (31). As some studies suggest, there is a strong link between psychological stress and cardiovascular diseases, with women having a higher sensitivity to the deleterious effects of stress system imbalances and stress hormone signaling (31, 32). They exhibit increased vascular reactivity to glucocorticoids, which could explain their elevated risk for stress-induced ischemia (33).

Previous studies have shown a significant association between stress-motivated eating and obesity, with women having a higher Body Mass Index (BMI) after stressful events compared to men (34, 35).

The influence of chronic stress on the HPA (hypothalamic-pituitary-adrenal) and HPO (hypothalamic-pituitary-ovarian) axes can have significant effects on reproduction. Hyperactivity of the HPA axis in response to prolonged stress results in hormonal imbalances, impacting body composition and insulin resistance. These changes, in turn, affect the functioning of the HPO axis, crucial for the maturation of reproductive organs and reproductive capacity. Changes in hormonal relationships, reduced oocyte competence, and impacts on fetal development may occur as a result of these alterations. Children of stressed mothers may present low birth weight, elevated anxiety, and dysfunction of the HPA axis (36–38). These studies highlight the numerous impacts of stress, especially in women.

Research, such as that conducted by Sangeetha et al. (26), suggests that expanding the investigation of IPV based on the Walker Cycle of Violence is a promising path to strengthen efforts to improve the wellbeing of women and eliminate the cycle of violence that makes them victims. Moreover, it raises awareness among women to leave abusive relationships and live independently. Therefore, it is crucial to consider the bidirectionality of violence and stress (7), where one can potentiate the other.

Thus, the combination of IPV and stress reveals a harmful cycle with disastrous impacts on health at various levels, from individual to societal (7). Despite the relevance of this phenomenon, the scarcity of studies on the topic, especially considering IPV throughout life and during the pandemic, limits the development of effective interventions and public policies for women exposed to violence, particularly in the context of Espírito Santo, Brazil. This gap represents an urgency in contemporary public health, and our pioneering study seeks to contribute to broader goals, such as the United Nations Sustainable Development Goals (SDGs) by 2030, promoting good health, wellbeing (Goal 3), and gender equality (Goal 5) to ensure the rights and health of women (39).

### 2.1 Objective of the research

To verify the association between perceived stress and the history of intimate partner violence throughout life and during the COVID-19 pandemic.

### 2.2 Research question

Is there a significant association between perceived stress and the history of intimate partner violence throughout life and during the COVID-19 pandemic?

## 3 Materials and methods

### 3.1 Study design and research location

This study is part of a broader study called “*Violência contra a mulherem Vitória, Esp*í*rito Santo: um estudo de base populacional*” (Violence against women in Vitória, Espírito Santo: a population-based study). This is an analytical cross-sectional epidemiological study carried out in the municipality of Vitória, Espírito Santo (ES), Brazil. This municipality had its population estimated for 2021 at 369,534 people, and the female population aged 18 years or older was about 155,673 (40). According to data from the last census, in 2010, the municipal Human Development Index was 0.845. The territory has 97.123 km^2^ (41).

#### 3.1.1 Participant consent

Prior to participation, explicit informed consent was obtained from all participants. This process, involving a written Informed Consent Form (ICF), ensured that participants were well-informed about the study's objectives and nature. The protection of anonymity was emphasized throughout. Interviews were conducted in participants' homes, in a private location, with only the interviewee and interviewer present, lasting an average of 30 min.

### 3.2 Period, population, and selection criteria

Data collection occurred between January and May 2022 and was performed by a team of properly trained female interviewers. Data were collected using tablets and managed with the assistance of the Research Electronic Data Capture (REDCap) electronic data capture tool. Field supervisors provided support to the team. The pilot study took place in December 2021, the data collected in it were not included in the final sample of the research. Fieldwork began after data analysis of the pilot study.

The sample was composed of women aged 18 years or older, living in the municipality of Vitória, with and without a history of violence (psychological, sexual, and/or physical) by an intimate partner. This was defined as the partner or ex-partner and/or current boyfriends if the couple maintains sexual relations, regardless of whether it was a formal union or not. Women who did not have the capacity to understand or communicate due to intellectual or sensory deficit and who, therefore, were unable to respond to the research data collection instruments were excluded from the study.

#### 3.2.1 Sampling process and sample definition

The sampling process occurred through multiple stages. The primary sampling unit was the census tracts of the municipality of Vitória provided by the 2010 Census conducted by the Brazilian Institute of Geography and Statistics (IBGE). The total number of households in the urban area of Vitória, Espírito Santo, in 2010 (108,515) was divided by 100 (the number of sectors to be visited) to obtain the systematic skip (1,085), ensuring proportional probability to the number of households and women within each sector.

Subsequently, the list was ordered by socioeconomic level, and the number 513 (between 1 and 1,085) was randomly selected using the R statistical program and Stata^®^ software version 15.1, corresponding to the number belonging to the first defined sector. The selection of the remaining sectors (99) proceeded by adding the systematic skip of the initial sector (184) and so on, successively until the end of the listing. Once the selection of census tracts was completed, the selection of households occurred randomly from the list available online by the IBGE. In each household, a list of eligible women, i.e., those meeting the study's inclusion criteria, was created, and one woman was then randomly chosen to respond to the interview.

For calculating the sample size of this study, the estimate of stress prevalence of 47.5% (42) was considered to maximize the sample, with a confidence level of 95%, acceptable error of 5%, plus 10% for losses and 30% for confounding factors, reaching a sample size of 1,100 women. For the present research, the sample consisted of 1,086 women.

### 3.3 Study variables

#### 3.3.1 Independent variables

The controlled variables were: sociodemographic data age/age group (18–29; 30–39; 40–49; 50–59; 60 or more), race/color (White; Black; Mixed race; Yellow, and Indigenous), years of schooling (0–8; 9–11; 12 or more), family income (1^st^tertile– poorer; 2^nd^tertile and 3^rd^tertile– richer), marital status (married; consensual union; single; separated or widowed), status of the house (own; rented or other), religion (no; yes), paid work (no; yes), government aid (no; yes), and number of residents in the household (living alone; 2; 3; 4; 5 or more people).

#### 3.3.2 Dependent variable

In this study, two outcomes were addressed: the levels of Perceived Stress among women who suffered IPV (psychological, sexual, and/or physical) throughout life (yes/no) and the levels of stress among women who suffered IPV during the COVID-19 pandemic (yes/no), that is, in the last 24 months prior to the research, which included the period of occurrence of the two waves of COVID-19 in Brazil (2020/2021) (43).

### 3.4 Instruments

Sociodemographic information was collected using a questionnaire that contained details specified in the independent variables. To screen for violence, the World Health Organization Violence Against Women (WHO VAW STUDY) (44) instrument was applied, translated and validated in Brazil, consisting of 13 questions, considering it present when the woman answered yes to one of the items for each type of VAW (psychological, sexual, or physical). To evaluate the Perceived Stress the short version of the Perceived Stress Scale (PSS) (15) was used, to evaluate the perceived stress regarding life experiences in the last month. The PSS is a Likert-type scale and has 10 questions with answer options ranging from zero to four (0 = never; 1 = almost never; 2 = sometimes; 3 = fairly often, and 4 = very often) for questions with negative connotations. On the other hand, questions with positive connotations have inverted scores (0 = 4, 1 = 3, 2 = 2, 3 = 1, and 4 = 0). The total of the scale is the sum of the scores of these 10 questions, and the score can range from 0 to 40. The scale, in Brazil, was translated and validated by Luft et al. (45) and its results are described in mean and standard deviation, a higher score indicates greater stress.

### 3.5 Analysis procedures of the study

The descriptive analyses of sample characterization were expressed in absolute and relative frequencies and their respective confidence intervals (95%CI) for categorical variables. The mean was also used as a measure of central tendency for variables with normal distribution plus their respective standard deviation. For the bivariate analyses, the following tests were used: *t*-test for means when we had independent variables in dichotomous form and analysis of variance (ANOVA) to verify the association and present the means by categories. The significance level was 5%. In the multivariate analysis, simple and multiple linear regression was used, with the betas (β) expressed in the tables. A significance level of 20% was used for entering the multivariable model and a significance level of 5% for remaining in the final model.

## 4 Results

### 4.1 Perceptions of stress among women who experienced intimate partner violence

#### 4.1.1 Throughout life

**Figure 1:**
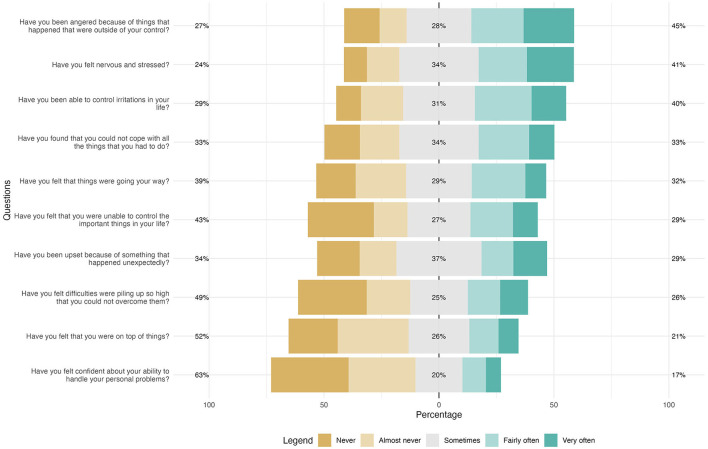
Perceived stress among women living in the municipality of Vitória who suffered intimate partner violence throughout their lives (N: 519). Vitória, ES, Brazil, 2022.

Figure 1 shows the perceptions of stress, in the last month, of women who suffered violence (sexual, psychological, and/or physical) by an intimate partner throughout their lives. According to the data presented, 29% of the women reported being sad due to unexpected events. Regarding the ability to control irritations in their lives, 29% responded negatively. When it comes to confidence in their ability to solve personal problems, 69% said they did not feel confident. Note that 20% reported feeling this confidence occasionally.

#### 4.1.2 During the pandemic

**Figure 2:**
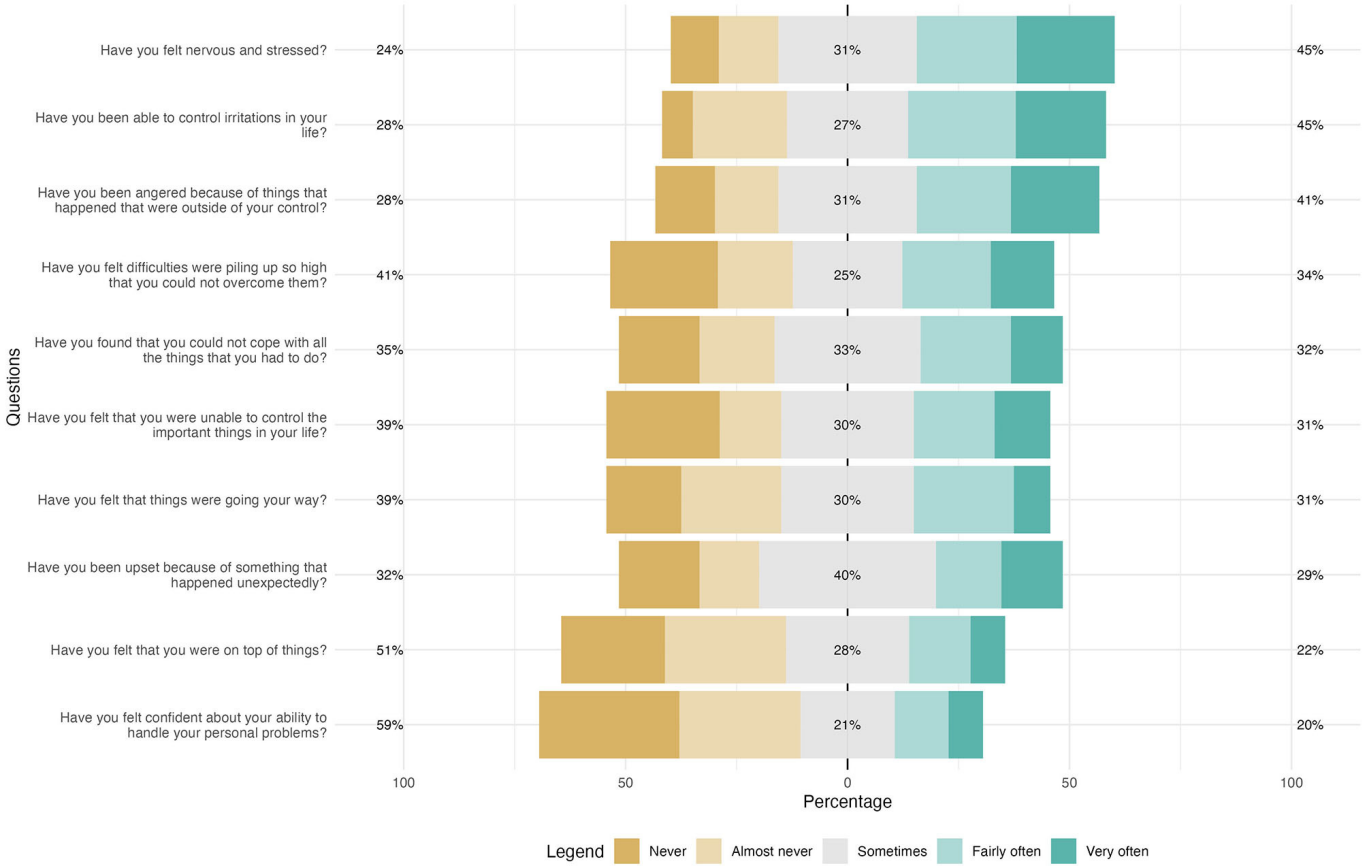
Perceived stress among women living in the city of Vitória who suffered intimate partner violence in the pandemic (last 24 months) (N: 231). Vitória, ES, Brazil, 2022.

Perceived stress (Figure 2), in relation to life experiences in the last month, of women who suffered violence (sexual, psychological, and/or physical) by an intimate partner during the pandemic (last 24 months) revealed that 32% of women were sad due to unexpected events. Regarding the ability to manage irritations in their lives, 28% reported not being able to do so. In addition, 59% of women did not feel confident in their ability to solve personal problems, with 21% reporting feeling this confidence occasionally.

### 4.2 Mean and standard deviation of perceived stress based on the presence of violence

#### 4.2.1 Throughout life

Women who suffered intimate partner violence in their lives had higher mean stress (18.49) when compared with those who did not experience this condition (14.49). Analyzing the three types of violence separately (psychological/emotional, physical, and sexual), the experience of each of them was associated with higher averages of perceived stress. Regarding violence throughout life, physical violence had the highest average (19.26), followed by sexual violence (19.25), with the latter being very close, and psychological/emotional (18.60) (*p* < 0.001) (Table 1).

**Table 1:** Means and standard deviation of perceived stress according to violence throughout life and during the pandemic among women aged 18 years or older living in the municipality of Vitória (N: 1,086).

**Violence throughout life and during the pandemic**	***N*^a^ (%)^b^**	**Mean**	**Standard deviation**	***p*-value**
**Violence throughout life**				< 0.001
No	567 (52.2%)	14.49	7.18	
Yes	519 (47.8%)	18.49	7.31	
**Psychological/emotional violence throughout life**				< 0.001
No	595 (54.8%)	14.59	7.19	
Yes	491 (45.2%)	18.60	7.30	
**Physical violence throughout life**				< 0.001
No	808 (74.4%)	15.42	7.34	
Yes	278 (25.6%)	19.26	7.25	
**Sexual violence throughout life**				< 0.001
No	899 (82.8%)	15.81	7.56	
Yes	187 (17.2%)	19.25	6.55	
**Violence during the pandemic**				< 0.001
No	855 (78.7%)	15.70	7.40	
Yes	231 (21.3%)	19.01	7.33	
**Psychological/emotional violence during the pandemic**				< 0.001
No	867 (79.8%)	15.76	7.42	
Yes	219 (20.2%)	18.95	7.32	
**Physical violence during the pandemic (*****N*** = **1,076)**				< 0.001
No	988 (91.0%)	16.11	7.51	
Yes	88 (9.0%)	19.32	6.89	
**Sexual violence during the pandemic**				< 0.001
No	1.015 (93.5%)	16.18	7.51	
Yes	71 (6.5%)	19.54	6.77	

Vitória, ES, Brazil, 2022. ^a^N, Raw frequency. ^b^%, Relative frequency.

#### 4.2.2 During the pandemic

Similarly, experiencing violence practiced by a partner during the period of isolation due to the COVID-19 pandemic was associated with higher mean stress (19.01) compared with women who did not experience this (15.70). In the context of the pandemic, there was a shift from physical violence (19.32) to sexual violence (19.54), with the latter now having the highest average. The physical violence was followed immediately by psychological/emotional (18.95) (*p* < 0.001) (Table 1).

### 4.3 Regression coefficients for perceived stress based on the occurrence of violence

#### 4.3.1 Throughout life

Table 2 shows that women who suffered violence by a partner in their lives presented, on average, 4 more points of stress than those who did not suffer violence, note that psychological/emotional violence stood out, also with an increase of 4 points, followed by physical violence with 3.8 and sexual violence with 3.4 (*p* < 0.001).

**Table 2:** Coefficients of simple regression of the relationship between the perceived stress among women living in the municipality of Vitória according to the occurrence of violence throughout life and during the pandemic (*N* = 1,086).

**Violence throughout life and during the pandemic**	***N*^a^ (%)^b^**	**β^c^**	**95%CI^d^**	***p*-value**
**Violence throughout life**				< 0.001
No	567 (52.2%)	Ref.		
Yes	519 (47.8%)	4.00	3.13–4.86	
**Psychological/emotional violence throughout life**				< 0.001
No	595 (54.8%)	Ref.		
Yes	491 (45.2%)	4.01	3.15–4.88	
**Physical violence throughout life**				< 0.001
No	808 (74.4%)	Ref.		
Yes	278 (25.6%)	3.84	2.84–4.84	
**Sexual violence throughout life**				< 0.001
No	899 (82.8%)	Ref.		
Yes	187 (17.2%)	3.44	2.27–4.61	
**Violence during the pandemic**				< 0.001
No	855 (78.7%)	Ref.		
Yes	231 (21.3%)	3.31	2.24–4.39	
**Psychological/emotional violence during the pandemic**				< 0.001
No	867 (79.8%)	Ref.		
Yes	219 (20.2%)	3.19	2.09–4.29	
**Physical violence during the pandemic (*****N*** = **1,076)**				< 0.001
No	988 (91.0%)	Ref.		
Yes	88 (9.0%)	3.20	1.65–4.75	
**Sexual violence during the pandemic**				< 0.001
No	1.015 (93.5%)	Ref.		
Yes	71 (6.5%)	3.35	1.55–5.15	

Vitória, ES, Brazil, 2022 ^a^N, Raw frequency. ^b^%, Relative frequency. ^c^β, Regression coefficient. ^d^95%CI, 95% confidence interval.

#### 4.3.2 During the pandemic

Similarly, victims during the pandemic recorded, on average, a 3.3 point increase in their stress levels compared with the group of non-victimized women. Sexual violence stood out in the significant increase in points in perceived stress, with 3.35 points, followed by physical violence with 3.2 and psychological/emotional violence, which also had an increase of 3.2 points (*p* < 0.001) (Table 2).

### 4.4 Multiple regression coefficients for perceived stress based on the occurrence of violence

#### 4.4.1 Throughout life

Table 3 shows the results obtained from the multiple linear regression analysis. Adjusting for sociodemographic variables, perceived stress remained associated with the occurrence of violence. Women who suffered violence from their intimate partner throughout their lives had, on average, 3.5 points more stress when compared with those who did not report this experience. Specifically, psychological/emotional violence stood out with the highest increase (β = 3.55), followed by physical violence (β = 3.49) and sexual violence (β = 3.08) (*p* < 0.001).

**Table 3:** Coefficients of the multiple regression of the relationship between the perceived stress among women living in the city of Vitória according to the occurrence of violence throughout life and during the pandemic (*N* = 1,086).

**Violence throughout life and during the pandemic**	***N*^a^ (%)^b^**	**β^c^**	**95%CI^d^**	***p*-value**
**Violence throughout life**				< 0.001^#^
No	567 (52.2%)	Ref.		
Yes	519 (47.8%)	3.54	2.69–4.39	
**Psychological/emotional violence throughout life**				< 0.001^#^
No	595 (54.8%)	Ref.		
Yes	491 (45.2%)	3.55	2.70–4.40	
**Physical violence throughout life**				< 0.001^#^
No	808 (74.4%)	Ref.		
Yes	278 (25.6%)	3.49	2.52–4.46	
**Sexual violence throughout life**				< 0.001^#^
No	899 (82.8%)	Ref.		
Yes	187 (17.2%)	3.08	1.95–4.22	
**Violence during the pandemic**				< 0.001^#^
No	855 (78.7%)	Ref.		
Yes	231 (21.3%)	2.83	1.78–3.88	
**Psychological/emotional violence during the pandemic**				< 0.001^#^
No	867 (79.8%)	Ref.		
Yes	219 (20.2%)	2.76	1.69–3.82	
**Physical violence during the pandemic (*****N*** = **1,076)**				0.001^#^
No	988 (91.0%)	Ref.		
Yes	88 (9.0%)	2.52	1.00–4.03	
**Sexual violence during the pandemic**				0.002^#^
No	1.015 (93.5%)	Ref.		
Yes	71 (6.5%)	2.73	0.99–4.48	

Vitória, ES, Brazil, 2022. ^a^N, Raw frequency. ^b^%, Relative frequency. ^c^ß, Regression coefficient.

^d^95%CI, Confidence interval. ^#^adjusted for age, self-declared skin color, years of schooling, income tertiles, marital status, having a religion, and number of residents in the household.

#### 4.4.2 During the pandemic

Similarly, victims during the pandemic showed, on average, 2.8 points more stress. Psychological/emotional violence also stood out in this context, with an increase of 2.76 points, followed by sexual violence with 2.7 and physical violence with 2.52 (*p* < 0.001) (Table 3).

Superior part of the form Superior part of the form.

## 5 Discussion

The results of this study pointed to an association between experiencing IPV throughout life and higher levels of stress in the women interviewed. On average, the perceived stress increased by 4 points in women who were victims of IPV. Even after considering sociodemographic factors, this association persisted, with an average increase of 3.5 points. These findings are consistent with previous studies (28, 29) that evidenced the impact of IPV on perceived stress.

The IPV is an overwhelming stressor, both emotional and personal, leading the victim to be constantly in a state of alert. A study points out that dealing with IPV is compared to going into “survival mode,” and the experience has been described as an overflowing glass (27). These findings corroborate the view that IPV is a unique and chronic stressor (7), reinforcing the importance of addressing it in a differentiated way in the analysis of its impacts on the stress perceived by the victims.

A systematic review conducted by Yim and Kofman highlighted that IPV can influence health by both biological pathways, related to endocrine and immunological aspects, and psychological pathways, such as stress. According to the review's findings, some dysregulations in endocrine and immunoinflammatory markers are associated with IPV, as well as psychological stress, which emerges in new cases of IPV. This suggests that IPV may contribute to increased stress in victims, and at the same time, high levels of stress may also increase the likelihood of IPV occurring or persisting (7).

Findings during the COVID-19 pandemic showed an average increase of 3.3 points in perceived stress among women victims of IPV, and 2.8 points after adjusting for sociodemographic variables. These results corroborate a study conducted in the United States, which aimed to identify differences in the levels of resilience and perceived stress between groups. That study revealed that victims of IPV reported lower resilience and higher perceived stress. These findings provide empirical support for asserting that public health measures adopted to combat the spread of COVID-19 can have negative and unintended impacts, such as increasing the risk of experiencing IPV, and the associated mental health outcomes (46).

During the pandemic, the disintegration of social and protection networks, women's lower contact with family and friends, limited access to services, prolonged contact with family members, possible economic or job losses, school closures, and increased workload related to family and child-care were factors that significantly increased stress and risk of violence for women (6).

Since the beginning of the pandemic, the incidence of Violence Against Women (VAW) has worryingly increased, which has been described as the “shadow pandemic” (47). In times of pandemic, the increase in gender-based violence may not be adequately addressed and given the attention it deserves. Previous experiences with epidemics such as Ebola and Zika have shown that such crises tend to exacerbate existing inequalities, including those related to gender and economic status (48). The crisis has disproportionately impacted women, highlighting that the effects of crises are never gender-impartial, and COVID-19 is no exception. Women have also been particularly affected by the economic and social outcomes of the pandemic, resulting from pre-existing inequalities in terms of economic position and social status (49).

Regarding the specific types of violence, be it psychological/emotional, physical, or sexual, in our study, all were related to an increase in the victims' perceived stress, both throughout life and during the pandemic. The IPV, in general, has adverse impacts on women's physical and mental health. In addition, over time, exposure to different types and multiple episodes of abuse appears to result in a cumulative detrimental effect. Another important point highlighted is the tendency for the frequent coexistence of various types of violence: physical IPV is often accompanied by sexual IPV and is often associated with emotional abuse (1), which can intensify the onset of traumatic symptoms and other health issues in affected individuals (50).

Thus, recognizing IPV as a unique and complex stressor, differentiating it from other chronic stressors, is essential. Factors such as the cycle of violence, the intensity and duration of IPV, the history of other experiences of abuse, the co-occurrence of other stressors, the possible resulting trauma, and the maintenance of an intimate relationship with the perpetrator of the violence (7) should be considered in studying this phenomenon. They can also allow for a deeper understanding of the impacts of IPV on victims' perceived stress.

## 6 Conclusion

The results of this study reveal a significant association between IPV throughout life and during the COVID-19 pandemic and increased levels of stress perceived by women. This association underscores the importance of carefully considering policies and interventions in times of crisis, such as the pandemic, since solutions adopted for one problem may inadvertently generate other challenges. Recognizing that this relationship is not just limited to periods of crisis, but permeates women's lives in ongoing and damaging ways, is crucial. In this context, in addition to addressing the implications of IPV during crises such as the pandemic, we must recognize the long-term impact of this violence.

Combining efforts in prevention and intervention to address this complex issue more comprehensively and carefully is essential. Promoting gender equality and preventing IPV should be guidelines from the early stages of life, aiming at deconstructing gender stereotypes and promoting healthy relationships. Preventing IPV cannot be as a momentary response, but rather as an enduring commitment to building a society that respects, protects, and empowers all women.

The findings of this study also highlight the urgency of developing effective, evidence-based interventions to address IPV and mitigate its negative effects on women's health. Promoting safe environments and adequate support for IPV victims should be a priority to ensure women's wellbeing and protection. Building a just and egalitarian society requires continuous and collaborative efforts, where prevention and intervention go hand in hand to address IPV and its devastating consequences.

Our study in this location is the first to propose the investigation of the association between intimate partner violence (IPV) throughout a woman's life and during the COVID-19 pandemic, and the perceived stress in women. To attain significant goals, such as the SDGs objectives until 2030–specifically, promoting good health and wellbeing (Goal 3) and gender equality (Goal 5) to safeguard the rights and health of women–it is imperative to first recognize the existing problems.

### 6.1 Limitations of the study

The importance of a careful evaluation of the results of this study is emphasized due to its cross-sectional nature, which prevents the inference of causality. However, the study found an association between exposure to IPV and the outcome perceived stress, providing information that can serve as a basis for future investigations, as well as careful evaluation with the victims and subsequent interventions. In addition, another limitation of this research refers to the information bias, focusing on the multiple issues related to the concept of violence that can influence the accuracy of the information provided by the participants, which can lead to a difficulty in measuring violence; however, as a way of mitigating this problem, note that the interview was conducted in a private space and with only the interviewee and the interviewer. Despite their limitations, analytical cross-sectional epidemiological studies strongly contribute to identifying possible associations between variables.

### 6.2 For further research

This research contributes to advance knowledge on the relationship between IPV and perceived stress, as well as it represents a basis for further research in future studies. Research with a longitudinal design to investigate variations in stress perceptions over time and possible associations between IPV and women's health is advised.

## Data availability statement

The raw data supporting the conclusions of this article will be made available by the authors, without undue reservation.

## Ethics statement

The research was approved by the Research Ethics Committee of the Federal University of Espírito Santo (CEP/UFES), with opinion number 4.974.080, and was registered under the Certificate of Presentation for Ethical Appreciation (CAAE) number 41628820.6.0000.5060. The studies were conducted in accordance with the local legislation and institutional requirements. The participants provided their written informed consent to participate in this study.

## Author contributions

LC: Conceptualization, Formal analysis, Investigation, Project administration, Validation, Visualization, Writing – original draft, Writing – review & editing. KM: Conceptualization, Supervision, Validation, Visualization, Writing – review & editing. FM: Conceptualization, Data curation, Funding acquisition, Investigation, Methodology, Supervision, Validation, Visualization, Writing – review & editing.
